# A report on the 2025 global conference on research and application of Chinese herbal medicine-derived extracellular vesicles

**DOI:** 10.20517/evcna.2025.157

**Published:** 2026-07-16

**Authors:** Zesheng Lu, Yingqi Cao, Wencong Shen, Zhengting Wu, Huijing Li, Dongxiao Li, Zimei Chen, Qing Zhao, Kewei Zhao

**Affiliations:** ^1^The Third Clinical Medical College of Guangzhou University of Chinese Medicine, Guangzhou 510375, Guangdong, China.; ^2^Guangdong Engineering Research Center of Chinese Herbal Vesicles, Guangzhou University of Chinese Medicine, Guangzhou 510375, Guangdong, China.; ^3^Guangzhou Key Laboratory of Chinese Medicine Research on Prevention and Treatment of Osteoporosis, The Third Affiliated Hospital of Guangzhou University of Chinese Medicine, Guangzhou 510378, Guangdong, China.; ^#^These authors contributed equally to this work.

**Keywords:** Chinese herbal medicine derived extracellular vesicles-like particles, plant-derived extracellular vesicles, extracellular vesicles, drug delivery, translational medicine, global conference

## Abstract

This conference report summarizes the Global Conference on Research and Application of Chinese herbal Medicine Derived Extracellular Vesicles-like Particles (CHM-EVLPs) held in Guangzhou. The conference gathered worldwide scholars to exchange cutting-edge advances in extracellular vesicle (EV) extraction, functional modification, disease treatment, and industrial transformation via six themed forums covering basic research, fruit EVLPs, laboratory testing, HIV intervention, and youth innovation. Experts shared standardized separation technologies, engineered modification strategies and multi-target therapeutic potentials of plant-derived extracellular vesicles (PDVs) against tumors, intestinal disorders, metabolic diseases and neurodegenerative injuries, while discussing key bottlenecks including large-scale production, quality control and regulatory guidelines. Cross-kingdom molecular communication, oral nano-delivery systems and interdisciplinary combinations with microneedles and artificial intelligence were highlighted as promising research directions. The event promoted international academic cooperation, clarified translational challenges of CHM-EVLPs products, and laid a foundation for advancing standardized, industrialized and globalized development of PDVs to facilitate worldwide medical care improvement.

## INTRODUCTION

To further promote the modernization and internationalization of traditional Chinese medicine (TCM), the 2025 Global Conference on Research and Application of Chinese herbal Medicine Derived Extracellular Vesicles-like Particles (CHM-EVLPs) was hosted in Guangzhou from August 8th to 10th, 2025. The event is jointly organized by the Guangdong Provincial Engineering Research Center of CHM-EVLPs, the Guangdong Provincial Association of Chinese Medicine, and the Third Affiliated Hospital of Guangzhou University of Chinese Medicine, with the support of the Professional Committee on Research and Application of extracellular vesicles (EVs) of the Chinese Society of Research Hospitals.

This conference, with the core theme of “Focusing on Medicinal plant-derived extracellular vesicles (PDVs) to Promote Global Pharmaceutical Development”, brought together top scholars and institutions from home and abroad. It was chaired by Professor Kewei Zhao, the director of the Guangdong Provincial Engineering Research Center of CHM-EVLPs. Through the main forum and five sub-forums covering basic research, fruit EVLP applications, integration of medical testing, exploration of human immunodeficiency virus (HIV) treatment, and youth innovation, it delved deeply into the cutting-edge breakthroughs in the field of CHM-EVLPs.

The conference attracted four hundred and fourteen participants and received support from the Guangdong Provincial Engineering Research Center of CHM-EVLPs, the Guangdong Provincial Association of Chinese Medicine, the Third Affiliated Hospital of Guangzhou University of Chinese Medicine, the Department of Medical Laboratory Science of the Third Affiliated Hospital of Guangzhou University of Chinese Medicine, the Professional Committee on Research and Application of EVs of the Chinese Society of Research Hospitals, the Professional Committee for Research and Application of CHM-EVLPs of the Guangdong Association of Chinese Medicine, Guangzhou University of Chinese Medicine School of traditional Chinese medicine, and the journal Interdisciplinary Medicine. The conference lasted for two days, with five renowned international scholars delivering keynote speeches. Fifty-three scholars gave presentations in the main forum and five sub-forums, respectively [Table 1]. Additionally, ten outstanding young scholars participated in the 2025 CHM-EVLPs Graduate Student Innovation Competition.

**Table 1:** Introduction of scholars attending the conference

**Name**	**Affiliation**	**Core academic title**	**Research field**
Main forum			
Qian Wang	The Professional Committee on Research and Application of EVs of the Chinese Society of Research Hospitals	Second-Class Professor, Doctoral Supervisor, and Distinguished Teacher of the National Ten-Thousand Talents Program	Precision diagnosis of cardiovascular diseases; EVs research
Frederic Zenhausern	University of Arizona, USA	Endowed Chair Professor (Tenured); Director of ANBM Center	Nano-biomedicine, drug delivery systems, personalized medicine
Hang Yin	School of Pharmaceutical Sciences, Tsinghua University	Professor, Former Vice Dean; Editor-in-Chief of Journal of *Extracellular Vesicle*	Exosome research and translation
Lei Zheng	Southern Hospital, Southern Medical University	Second-Class Professor, Doctoral Supervisor; Vice President; National Outstanding Young Scientist Fund Recipient	New technologies and markers for *in vitro* diagnosis of major diseases
Liwen Jiang	School of Life Sciences, The Chinese University of Hong Kong	Choh-Ming Li Professor; Director of Research Centers	Protein transport, organelle biogenesis
Kewei Zhao	The Third Affiliated Hospital of Guangzhou University of Chinese Medicine	Chief Technologist, Doctoral Supervisor; Director of Clinical Laboratory	CHM-EVLPs research, molecular diagnostics
Haifang Yin	Medical Technology Department, Tianjin Medical University	Professor, Doctoral Supervisor; Dean; National High-Level Talent Program Leader	Non-invasive diagnosis, targeted therapy, exosome application
Seppo J. Vainio	The University of Oulu, Finland	Professor of Developmental Biology; Scientific Director of Kvantum Institute	EVs-based diagnostics/therapeutics, regenerative medicine
Qiang Cai	College of Life Sciences, Wuhan University	Professor, Doctoral Supervisor; Chairman of Hubei Plant Physiology and Molecular Biology Society	Vesicle-mediated cell communication in plant-pathogen interactions
Weiliang Xia	Shanghai Jiao Tong University	Tenured Full Professor, Doctoral Supervisor; Vice Dean of Zhiyuan College	Tumor exosome biology and diagnosis/treatment
Zifeng Yang	Guangzhou Institute of Respiratory Health	Researcher, Vice President; PI of National Key Laboratory	Integrated TCM-WM for respiratory diseases; Antiviral drug R&D
Jianxin Wang	School of Pharmacy, Fudan University	Second-Class Professor, Doctoral Supervisor; Secretary of Party Committee	Novel drug delivery systems; TCM preparation R&D
Quanxi Mei	The Third Affiliated Hospital of Guangzhou University of Chinese Medicine	Professor, Chief Pharmacist, Doctoral Supervisor; Academic Leader of Pharmacy Department	TCM preparation development; TCM standardization
Anggraini Barlian	The School of Life Sciences and Technology, Bandung Institute of Technology, Indonesia	Researcher	Cell & developmental biology; Stem cells & exosomes; PDVs research
Yunyao Jiang	School of Pharmaceutical Sciences, Tsinghua University	Researcher	TCM pharmacology; Astragalus EVLP; Intestinal injury repair
Zhangfeng Zhong	Institute of Chinese Medicine, University of Macau	Associate Professor, Doctoral Supervisor; Independent Principal Investigator	Development of medicinal and edible homologous Chinese medicinal products; PDVs R&D
Jinghua Chen	Fujian Medical University	Professor, Doctoral Supervisor; Dean of Graduate School	Drug delivery systems; Nanobiomedicine
Bo Xiao	Sichuan Provincial People’s Hospital, Affiliated Hospital of University of Electronic Science and Technology of China	Professor	Oral nano-drug systems; Plant lipid vesicles
Chao Wang	Soochow University	Professor, PhD	Immune-regulating bio-nanomaterials; Immunotherapy
Yinghua Peng	Institute of Special Commodities, CAAS	Researcher, Doctoral Supervisor; Chief of CAAS Team	Functional factors of special animals/plants; Nutrition and health regulation
Jun Wu	HKUST	Professor; Vice President of Systems Hub, HKUST (Guangzhou)	Biomaterials; Drug-controlled release; Tissue engineering; Nanomedicine
Bin Tean Teh	Duke-NUS Medical School of Singapore Health Group	Professor; Deputy CEO of National Cancer Centre Singapore	Cancer genomics; Translational medicine
Guojun Zhang	Hubei University of Chinese Medicine	Second-Class Professor, Doctoral Supervisor; Dean of School of Laboratory Medicine	Analytical chemistry; CHM-EVLPs microneedle drug delivery systems
Zhenhua Li	The Tenth Affiliated Hospital of Southern Medical University	Researcher, Doctoral Supervisor; National Outstanding Young Scientist Fund Recipient	Biomaterials-based tumor therapy; Myocardial tissue engineering; EVs engineering
Jiawen Wang	Changchun University of Chinese Medicine	Senior Researcher, Doctoral Supervisor; Jilin Provincial High-Level Talent (Class D)	TCM traditional efficacy mechanism; Ginseng EVLPs research
Basic Research on CHM-EVLPs			
Ling Li	Hunan University of Chinese Medicine	Professor, MD, Doctoral Supervisor; Director of Changsha Engineering Technology Innovation Center for CHM-EVLPs	Natural medicine anti-infection and immunoregulation; Nano-delivery systems
Chaoxiang Chen	Jimei University	Associate Professor; Fujian Provincial High-Level Talent	Food EVLP isolation/purification; Functional modification; Targeted therapy for tumors/obesity/aging
Huantian Zhang	The First Affiliated Hospital of Jinan University	Professor, Chief Physician, Doctoral Supervisor; Deputy Director of Orthopedics Department	Joint barrier imbalance; Osteoarthritis mechanism; Artificial intelligence/robot-assisted surgery
Fengxi Yang	Institute of Environmental Horticulture, Guangdong Academy of Agricultural Sciences	Researcher, PhD; Director of Institute; Chief Expert of Flower Innovation Team	Orchid germplasm resource innovation; Medicinal function of orchids; EVs application
Lifu Wang	Guangzhou Medical University	Professor, Doctoral Supervisor; Deputy Secretary-General of Guangdong Society of Parasitology	Exosome-mediated pathogen-host interaction; CHM-EVLPs for enteritis treatment
Han Liu	Shanghai University Institute of Translational Medicine	Associate Researcher; Director of EVs Research Center	EVs research; Bone aging transformation research
Research and application of EVLP derived from fruits			
Junbing Fan	School of Basic Medical Sciences, Southern Medical University	Professor, Doctoral Supervisor; Deputy Director of Institute of Oncology	Biomimetic materials (EVs/ECM); Tumor drug delivery; Organ regeneration
Suhua Qi	Xuzhou Medical University	Professor, Doctoral Supervisor; Director of Xuzhou Key Laboratory of Clinical Laboratory Diagnosis	Cardiovascular & cerebrovascular disease mechanism; Drug screening; TCM-derived EVs regulating glucose metabolism
Wei Yang	Tongji Medical College, Huazhong University of Science and Technology	Associate Professor, PhD; Deputy Director of Environmental and Health Research Platform	Nutrition & food hygiene; wolfberry EVLP for myocardial infarction repair
Xiaozhou Mu	Clinical Medical Research Institute, Zhejiang Provincial People’s Hospital	Researcher, Professor, Doctoral Supervisor; Executive Deputy Director of Clinical Medicine Research Institute	Medicinal PDVs; Oncolytic virus development; Targeted delivery
Xiaohong Yang	Chongqing Institute of Green and Intelligent Technology, Chinese Academy of Sciences	Associate Researcher, PhD	Engineered exosomes for anti-infection; Exosome purification technology
Xiangsheng Cai	Guangzhou Eleventh People’s Hospital	Associate Researcher, MD	Chronic disease treatment with stem cells/CHM-EVLPs; Engineered EVs development
CHM-EVLPs and medical laboratory testing			
Sergio Bernardigi	University of Rome Tor Vergata	Full Professor of Clinical Biochemistry & Molecular Biology; Director of Clinical Molecular Biology Department	Pediatric endocrinology; Oncology (neuroblastoma); Neurodegenerative diseases
Qi Li	Xiyuan Hospital, China Academy of Chinese Medical Sciences	Chief Technologist, MD, Master Supervisor; Director of Clinical Laboratory and Blood Transfusion Department	TCM integrated laboratory diagnosis; Medical laboratory accreditation
Canyun Xu	Ji’an Central People’s Hospital	Associate Professor, MD, Master Supervisor	Clinical immune disease marker screening; Neuroprotective mechanism of cordycepin
Zhongfang Wang	Guangzhou National Laboratory	Professor, Researcher, Doctoral Supervisor; Vice President of Hetao Research Institute; Director of Detection and Evaluation Center	Clinical medicine; Immunology; Virology (Respiratory virus infection immunity)
Yuanchen Ma	Guangdong Provincial People’s Hospital	Chief Physician, MD, Master Supervisor; Deputy Director of Joint Surgery	Orthopedics; VTE prevention and treatment; Thrombus markers
Xiangdong Kang	Shanghai University of Traditional Chinese Medicine	Professor, Chief Technologist, Doctoral Supervisor; Director of Clinical Laboratory	TCM theoretical basis of tumors; Experimental research
Honghai Hong	Guangzhou Medical University Third Affiliated Hospital	Associate Chief Technologist, Associate Researcher, MD, Master Supervisor	Tumor apoptosis mechanism; Tumor marker screening; Alzheimer’s disease pathogenesis
CHM-EVLPs and HIV			
Tong Wang	College of Life Science and Technology, Jinan University	Researcher, Second-Class Professor, Yangtze River Scholar Distinguished Professor	HIV/ hepatitis B detection technology; EVs-based drug resistance testing
Xin Deng	Guangxi University of Chinese Medicine	Second-Class Professor, Doctoral Supervisor, National Young Qi Huang Scholar	TCM treatment of infectious diseases; Geriatric AIDS
Ying Liu	AIDS Center of China Academy of Chinese Medical Sciences	Researcher, Doctoral Supervisor	TCM prevention and treatment of AIDS
Weihua Li	Beijing You’an Hospital, Capital Medical University	Researcher, MD, Professor, Doctoral Supervisor; Director of Office of Beijing Institute of Integrated TCM and WM	TCM treatment of AIDS; Exosome mechanism research
Luping Lin	The Eighth Affiliated Hospital of Guangzhou Medical University	Chief Physician, MD, Master Supervisor; Director of TCM Department	Integrated TCM-WM treatment of AIDS; Emerging infectious diseases
Youth forum			
Zhiguo Zhang	Institute of Basic Theory of TCM, China Academy of Chinese Medical Sciences	Researcher, Doctoral Supervisor	TCM prevention and treatment of chronic diseases; *Pulsatilla chinensis* EVLP for ulcerative colitis
Yali Liu	Nanjing University of Chinese Medicine	Associate Professor	Transdermal treatment of CHM-EVLPs; Psoriasis
Tianxin Qiu	Third Affiliated Hospital of Guangzhou University of Chinese Medicine	Postdoctoral Fellow, Associate Researcher	*Pueraria lobata* EVLP in Preventing and Treating Alcoholic Liver Disease Based on the “Gut-Liver Axis”
Jiayang Gao	The Chinese University of Hong Kong	Postdoctoral Fellow	Morphology and function of plant EVs

ANBM: Arizona Network for Biomedical Research; CAAS: Chinese Academy of Agricultural Sciences; CHM: Chinese herbal medicine; CHM-EVLPs: Chinese herbal medicine-derived extracellular vesicle-like particles; ECM: extracellular matrix; EVs: extracellular vesicles; EVLP: extracellular vesicle-like particle; HKUST: The Hong Kong University of Science and Technology; HIV: human immunodeficiency virus; MD: Doctor of Medicine; PDVs: plant-derived vesicles; PhD: Doctor of Philosophy; PI: principal investigator; R&D: research and development; TCM: traditional Chinese medicine; VTE: venous thromboembolism; WM: Western medicine.

During this conference, the experts present demonstrated a naturally harmonious atmosphere of communication, jointly creating a pleasant and efficient environment. We would like to express our gratitude to all the participants, the organizing team, and the support staff for their hard work, which ensured the smooth running of the conference. Next, we will report on the experts’ wonderful speeches and summarize the core content of the conference.

## PART 1 MAIN FORUM

**Professor Qian Wang** (the Professional Committee on Research and Application of EVs of the Chinese Society of Research Hospitals) shared the progress of the professional committee since its establishment in 2017, with the core focus on promoting the translation of EVs from basic research to clinical application. He pointed out that EVs, as key cellular messengers, have great potential in disease diagnosis and treatment such as tumors and tissue repair, and particularly introduced the characteristic direction of CHM-EVLPs that integrates TCM with modern science. Facing existing challenges such as the standardization of separation techniques and detection methods, Professor Wang called for strengthened international cooperation to jointly promote the standardization of EVs research and its clinical application.

**Professor Frederic Zenhausern** (University of Arizona, USA) systematically introduced the research progress of his team in the field of PDVs. He showcased the advantages of the University of Arizona in medicine, drug delivery, and bioengineering, and emphasized the importance of research infrastructure and interdisciplinary collaboration. He focused on the great potential of PDVs as drug-delivery carriers, which have excellent biocompatibility, drug-loading capacity, and low immunogenicity. In response to current challenges such as standardized production and batch stability, he proposed solutions through bioengineering modification and vertical agricultural technology, and called for strengthened international cooperation to accelerate their clinical transformation and industrialization.

**Professor Hang Yin** (School of Pharmaceutical Sciences, Tsinghua University) delivered a keynote speech on “Research and Transformation of Exosomes”. He highlighted the research progress of the team regarding quality standards for traditional Chinese medicinal materials. By applying omics technologies, the team identified significant quality variations among traditional Chinese medicinal materials from different geographic origins and ages. Furthermore, the team is actively contributing to the development of International Organization for Standardization (ISO), thereby promoting the global standardization and internationalization of TCM. Professor Yin pointed out that EVs have broad application prospects in cell communication, medical aesthetics, anti-aging, and the prevention and treatment of neurodegenerative diseases. He called for strengthened academic cooperation to promote the industrialization and internationalization of CHM-EVLPs with Chinese characteristics.

**Professor Lei Zheng** (Southern Hospital, Southern Medical University) pointed out that CHM-EVLP is a powerful new approach to address the issues of TCM, such as toxic and side effects, poor targeting, and low bioavailability. He emphasized the multifunctionality of CHM-EVLPs in disease treatment and introduced a reconstructed curcumin EVLP combined with biomaterials developed by his team, which has shown great potential in the treatment of diabetic foot and atopic dermatitis. Professor Zheng believes that the rich experience of TCM provides a direction for CHM-EVLPs research, and in the future, the potential of compound application should be explored. However, there are still challenges such as the need to optimize separation and purification technology, the lack of large-scale preparation and standardization systems, and the mechanism of action and pharmacological effects still need to be further clarified through effective labeling and other techniques.

**Professor Liwen Jiang** (School of Life Sciences, The Chinese University of Hong Kong) delved deeply into the formation mechanism, structure and function of PDVs. He pointed out that PDVs mainly originate from the double-membrane organelle named extracellular protein output (EXPO), which forms through a non-classical pathway of “outer membrane-plasma membrane fusion and release of the inner membrane”, and needs to overcome the cell wall barrier for transport. Functionally, PDVs play a significant role in plant defense responses and growth and development regulation by carrying specific signaling molecules. Professor Jiang’s team utilized three-dimensional electron microscopy technology to clearly reveal the dynamic structure of PDVs and their transmembrane transport capabilities, providing a powerful tool for understanding intercellular communication in plants. Despite the great application potential of PDVs in agriculture and biotechnology, the precise molecular mechanism of action and regulatory network remain the core challenges for future research.

**Professor Kewei Zhao** (The Third Affiliated Hospital of Guangzhou University of Chinese Medicine) presented the latest progress on CHM-EVLPs as an emerging drug delivery system and its potential in promoting the modernization and internationalization of TCM. He pointed out that CHM-EVLPs, as a new drug delivery system, have advantages such as multi-component synergy, efficient targeting, and safety. Professor Zhao demonstrated the rapid development of CHM-EVLPs research through the publication data over the past 10 years, covering areas such as drug delivery, anti-tumor, and inflammation regulation. He also emphasized that current industrialization still faces challenges such as extraction and purification, stability control, and cost, especially the incomplete quality standard system, which urgently needs to establish a high-standard quality control system. In the future, his team will focus on the standardization of CHM-EVLPs technology, its mechanism of action, and industrial transformation, promoting its transition from the laboratory to clinical and practical applications.

**Professor Haifang Yin** (Medical Technology Department, Tianjin Medical University) presented the latest research progress by her team in the extraction, functionalization, and anti-tumor application of CHM-EVLPs. To address the limitations of the traditional grinding method, such as low yield and insufficient purity, the team developed an enzymatic digestion method. By optimizing reaction parameters and combining ethanol rinsing with antibiotic treatment, a standardized and sterile extraction process was established. Proteomics and lipidomics analyses indicated that this method could well preserve the bioactive components and functions of the EVs. In terms of functional application, the team employed nebulization delivery and functionalization approaches to demonstrate that *Tripterygium wilfordii* EVLP can effectively suppress tumor growth in non-small cell lung cancer models. Notably, when administered in combination with cisplatin or loaded with antigens and anti-PD-1 antibodies, it significantly enhanced CD8+ T cell infiltration and prolonged mouse survival. Future research will focus on optimizing the internal functionalization of *Tripterygium wilfordii* EVLP to advance its therapeutic potential in oncology.

**Professor Seppo J. Vainio** (The University of Oulu, Finland) introduced the nutritional extracellular vesicle (NutriEV) project, highlighting its promising applications in targeted therapy and non-invasive biosensors. He highlighted that NutriEV exhibits excellent targeting capability and biocompatibility, enabling effective penetration of the blood-brain barrier for the treatment of neurodegenerative diseases, as well as non-invasive delivery via aerosol inhalation. These vesicles can be derived from natural foods or cell cultures, offering high purity and straightforward functional customization. Additionally, Professor Vainio highlighted the potential of EVs in biosensing applications, noting that changes in EVs present in bodily fluids such as saliva and urine can be leveraged for non-invasive health monitoring. As an interdisciplinary initiative integrating food science, biotechnology, and medicine, NutriEV aims to advance the innovative application of EVs in agriculture and medicine.

**Professor Qiang Cai** (College of Life Sciences, Wuhan University) presented the complex mechanism of cross-kingdom interaction between plants and pathogenic bacteria through EVs. PDVs not only transport microRNAs (miRNAs) to inhibit the expression of pathogenic bacteria genes but also deliver disease-resistant proteins to enhance plant disease resistance. Meanwhile, EVs produced by pathogenic bacteria suppress plant immune responses through the delivery of effector proteins, highlighting the sophisticated interplay between plants and pathogenic bacteria in cross-kingdom RNA and protein transport. Moreover, catalase present in bacterial EVs can reduce reactive oxygen species (ROS) levels in plants, thereby facilitating bacterial survival within host tissues. These findings by Professor Cai provide critical scientific evidence for understanding the functional roles of PDVs in cross-kingdom molecular transport and their involvement in plant defense mechanisms and bacterial pathogenicity.

**Professor Weiliang Xia** (Shanghai Jiao Tong University) introduced the characteristics, functions, and progress in applications of PDVs. He pointed out that as cross-regulatory carriers, PDVs have advantages such as wide sources, high uptake efficiency and good biocompatibility, and have shown good therapeutic effects in models of tumors, metabolic diseases and intestinal flora imbalance. In addition, his team’s research demonstrated that celery EVLP not only exhibits higher cellular uptake efficiency and superior drug-delivery capability compared to liposomes but also effectively suppresses programmed death-ligand 1 (PD-L1) expression synergistically. Professor Xia also highlighted that the field continues to face challenges in large-scale separation and purification, batch-to-batch consistency, surface labeling, identification of active components, cross-regulatory mechanisms, and the establishment of clinical safety standards. Finally, as Associate Editor of the journal “Extracellular Vesicles and Circulating Nucleic Acids”, Professor Xia invited researchers in the academic community to submit their work, fostering open communication and advancing the in-depth development of this field.

**Professor Zifeng Yang** (Guangzhou Institute of Respiratory Health) reviewed the history of drug development in China, particularly the research on anti-influenza drugs based on TCM such as Isatidis Radix. He pointed out that China’s drug development has advanced from conventional chemical drugs to antiviral agents, exemplified by the use of neuraminidase inhibitors, yet challenges such as drug resistance persist. Professor Yang’s team targeted the novel Polymerase Basic Protein 2 (PB2) and successfully developed a new anti-influenza drug with independent intellectual property rights, marking a significant breakthrough in the field. He emphasized that drug research and development must be closely aligned with clinical needs and market-oriented transformation. It is essential to leverage multidisciplinary collaboration and the national innovation system to accelerate breakthroughs, and to promote the translation of research outcomes through an integrated review mechanism linking scientific research, clinical application, and regulatory oversight. He further called for enhanced global cooperation to facilitate the internationalization of Chinese medicines, reflecting China’s open vision and growing responsibility in the global landscape of new drug development. Moreover, his study provides insights into the antiviral applications of CHM-EVLPs.

**Professor Jianxin Wang** (School of Pharmacy, Fudan University) has developed a novel separation method based on Tim-1 protein-functionalized magnetic nanoparticles, which can efficiently and specifically isolate PDVs from plants. Compared with the traditional ultracentrifugation method, this method takes less time, has higher purity, and can better preserve the active components of PDVs. The research further used the obtained PDVs to load nerve growth factor, and with the help of magnetic targeting and external magnetic field guidance, significantly improved the efficiency of the drug crossing the blood-brain barrier. In a mouse model of cerebral ischemia, this system demonstrated multiple therapeutic effects, including reducing the area of cerebral infarction, promoting the differentiation of neural stem cells, and improving neurological function, and also had MRI imaging capabilities, achieving the integration of diagnosis and treatment. Professor Wang’s research provides new ideas for the extraction, drug loading, and clinical transformation of PDVs.

**Professor Quanxi Mei** (The Third Affiliated Hospital of Guangzhou University of Chinese Medicine) pointed out that the regulatory bottleneck faced by CHM-EVLPs in clinical application lies in the fact that its nature has not been clearly defined by current regulations, which leads to its inability to pass ethical review. He proposed two paths. One is to adopt the model of TCM ad hoc preparations, which are exempt from approval based on the “one prescription for one individual” principle; the other is to apply for hospital preparations for compound or complex dosage forms. Professor Mei called on the industry to quickly formulate consensus norms for clinical research on CHM-EVLPs and urged the drug regulatory authorities to intervene and establish application guidelines to promote the development of the industry.

**Professor Anggraini Barlian** (The School of Life Sciences and Technology, Bandung Institute of Technology, Indonesia) introduced their research on PDVs at Bandung Institute of Technology. She pointed out that PDVs have the advantages of high safety, abundant raw materials, and much lower costs than stem cell exosomes. They utilized the rich local herbal resources to establish a standardized separation and purification process, and confirmed that PDVs from ginger, beans, *Centella asiatica*, *etc*. have excellent anti-inflammatory, chondroprotective, and wound healing activities. The team developed a freeze-dried hydrogel formulation, which enables room-temperature storage and transportation as well as controlled release, and has no cytotoxicity. Professor Barlian also mentioned that PDVs can target deliver miRNA and promote collagen synthesis, and can be applied in anti-inflammatory, anti-cancer, medical aesthetics, and functional food fields.

**Professor Yunyao Jiang** (School of Pharmaceutical Sciences, Tsinghua University) presented the multi-level research conducted by his team, revealing the significant role of *Astragalus membranaceus* EVLP in anti-aging, anti-liver fibrosis, treating coronary heart disease, and protecting against intestinal injury. His team used the “boiling method” to isolate EVLP from *Astragalus membranaceus* and confirmed that it can specifically accumulate in the colon after oral administration. In a sleep deprivation mouse model, *Astragalus membranaceus* EVLP effectively reversed colon shortening, crypt destruction, inflammatory infiltration, and microbiota dysbiosis, with better effects than traditional extracts. Multi-omics analysis further clarified that its mechanism of action stems from the miRNAs carried by *Astragalus membranaceus* EVLP, which can restore intestinal barrier function and amino acid homeostasis by inhibiting amino acid transporters and upregulating tight junction proteins. This study provides a new therapeutic perspective and mechanistic insight for the application of CHM-EVLPs in intestinal injury caused by sleep deprivation.

**Professor Zhangfeng Zhong** (Institute of Chinese Medicine, University of Macau) systematically introduced the research progress of his team in the treatment of ulcerative colitis with *Centella asiatica* EVLP. He pointed out that compared with TCM dosage forms, CHM-EVLPs have significant advantages in retaining biological activity and enhancing the safety of medication, and have become an important direction for the modernization of Chinese medicine. The team successfully extracted and characterized *Centella asiatica* EVLP by ultracentrifugation, with a particle size of about 150 nanometers and rich in proteins and lipids. The research further screened out key functional miRNAs and verified their important role in alleviating colonic inflammation. To improve the therapeutic effect, the team developed a calcium-dopamine modification strategy, which significantly enhanced the stability and anti-inflammatory effects of *Centella asiatica* EVLP in the gastrointestinal environment, providing a new path for the engineering modification of CHM-EVLPs. In addition, Professor Zhong looked forward to their industrialization prospects and planned to develop dosage forms such as microneedles and facial masks based on CHM-EVLPs to promote the application of such preparations from research to practical use.

**Professor Jinghua Chen** (Fujian Medical University) presented his team’s innovative research on the functional remodeling of CHM-EVLPs. Addressing the three core challenges of difficult PDV extraction, low drug loading rate, and weak targeting, the team, from an analytical chemistry perspective, successfully prepared uniform-sized aloe EVLPs by precisely regulating medium viscosity and centrifugation parameters. The research found that low osmotic salt stress could prompt medicinal plants to spontaneously enrich indocyanine green (ICG) and simultaneously remodel the lipid membrane structure of EVs, significantly reducing liver retention and enhancing stability. In terms of targeting strategies, the team proposed using quantum chemical calculations to guide molecular co-loading and replacing antibodies with DNA aptamers to achieve precise tumor enrichment. Additionally, Professor Chen introduced the study on cucumber EVLP carrying momordicine to reverse doxorubicin resistance by inhibiting signal transducer and activator of transcription 3 (STAT3) phosphorylation. He emphasized that in the future, it is necessary to systematically integrate the synergistic effects of external loads and internal active components of EVs and optimize their functions through biological or non-biological stress strategies. At the same time, the introduction of artificial intelligence (AI) and computational simulation technologies will further enhance the application efficiency of PDVs in the treatment of diseases such as cancer.

**Professor Bo Xiao** (Sichuan Provincial People’s Hospital, Affiliated Hospital of University of Electronic Science and Technology of China) introduced the application of oral nanomedicine systems based on PDVs for the treatment of colonic diseases. Leveraging the exceptional gastrointestinal stability and biocompatibility of PDVs, the team developed two delivery strategies. The first involved encapsulating a gene-editing system targeting lysine-specific demethylase 1 (LSD1), enabling efficient gene editing and significant anti-inflammatory effects in animal models. The second strategy utilized a chemical conjugation approach to link probiotics to the system, allowing their targeted release in the tumor microenvironment and achieving dual therapeutic effects: immune activation and photothermal/photodynamic tumor ablation. Furthermore, the research demonstrated that this system could specifically target macrophages, exhibit strong mucus penetration capability, and exert potent anti-tumor and anti-metastatic effects in colon cancer models. These findings reveal the multifaceted mechanisms underlying oral nanomedicine systems and highlight the advantages of integrating multidisciplinary approaches in therapeutic development.

**Professor Chao Wang** (Soochow University) reported an oral nano-immunotherapy based on garlic EVLP. The research team used a self-developed process to scale up the production of garlic EVLP and confirmed that it specifically accumulates in the intestine after oral administration. It directly activates γδ T cells at the nanoscale, promoting their proliferation and differentiation into interferon-gamma positive (IFN-γ^+^) anti-tumor phenotypes. These cells then migrate to tumor sites along the CXCL10-CXCR3 axis, reshaping the immune microenvironment by secreting IFN-γ and effectively inhibiting various subcutaneous tumors in mice. Mechanistically, the study identified γδ T cells as the key effector cells and demonstrated a synergistic effect when combined with a PD-L1 antibody. This strategy offers both safety and cost-effectiveness advantages, providing a new approach for tumor immunotherapy.

**Professor Yinghua Peng** (Institute of Special Commodities, Chinese Academy of Agricultural Sciences) systematically expounded on the multiple roles of *Platycodon grandiflorum* EVLP in reversing immunosuppression and reshaping the intestinal microbiota. The team successfully isolated *Platycodon grandiflorum* EVLP with high phospholipid content, which exhibits excellent stability and efficient cellular uptake. The study demonstrates that *Platycodon grandiflorum* EVLP effectively inhibits the proliferation of breast and lung cancer cells and induces apoptosis *in vitro*. *In vivo*, they significantly suppress tumor growth following oral or intravenous administration. The underlying mechanisms involve promoting tumor cell apoptosis, inhibiting angiogenesis, inducing M1 macrophage polarization, and downregulating PD-L1/PD-1 expression, thereby enhancing T cell infiltration and anti-tumor immune responses. Furthermore, *Platycodon grandiflorum* EVLP can modulate the gut microbiota, thereby improving the tumor microenvironment and synergistically augmenting therapeutic efficacy.

**Professor Jun Wu** (The Hong Kong University of Science and Technology) shared his research experience and innovative ideas in the field of engineering EVs and their biomedical applications, focusing on the areas of degradable biomaterials, drug carriers, and tissue-engineering scaffolds. He emphasized the importance of developing multifunctional organic biomaterials, particularly carrier systems that integrate drug delivery with intrinsic therapeutic functions. His research combines natural and synthetic approaches, encompassing the construction of polyester libraries derived from bioactive monomers in TCM - such as ferulic acid, ursolic acid, and salicylic acid - the development of food-medicine homologous materials, and the fabrication of novel liposomes and exosomes for applications in treating tumors, cardiovascular diseases, and inflammatory conditions. Furthermore, his team has explored the potential of food-medicine homologous materials in regenerative medicine, demonstrating their significant efficacy in wound healing and bone regeneration. In addition, the research incorporates AI-assisted screening of active TCM monomers, offering innovative pathways for functional advancement of biomaterials and addressing complex medical challenges.

**Professor Bin Tean Teh** (Duke-NUS Medical School of Singapore Health Group) delivered a speech titled “Biodiversity Research Translating for Health and Medical Development”, introducing the Singapore Centre for Ecological Medicine that he leads. The center is dedicated to transforming plant research into applications that promote human health. Professor Teh shared the breakthroughs of his team in tumor genetics, durian genome, and orchid research, demonstrating the great potential of plants in drug development and nutrition. Additionally, the team has established vertical farms and herb gardens to systematically study the nutritional value of plants and their positive impact on patients’ health. Professor Teh stated that the team hopes to focus on advancing the research of PDV engineering, aiming to develop more sustainable health solutions for patients and the public.

**Professor Guojun Zhang** (Hubei University of Chinese Medicine) introduced the innovative application of the *Coptis chinensis* EVLP microneedle drug delivery system in wound healing. Coptis chinensis, as a TCM, has significant antibacterial and anti-inflammatory effects, but its direct use may cause skin irritation. To address this issue, the research team developed the *Coptis chinensis* EVLP microneedle system, which uses CHM-EVLPs as a drug carrier and delivers the drug to the deep layers of the wound through microneedle technology, reducing bacterial load, promoting wound healing, and alleviating inflammatory responses. Professor Zhang also demonstrated the application potential of lemon EVLP in tumor treatment and emphasized that in the future, they will further combine TCM with modern technology to promote the application of CHM-EVLPs in the medical field.

**Professor Zhenhua Li** (The Tenth Affiliated Hospital of Southern Medical University) presented research progress on PDVs and their engineered preparation techniques. He pointed out that, compared to animal EVs, which involve complex preparation procedures and exhibit limited efficiency, PDVs have emerged as a new research hotspot owing to their high yield and straightforward production process. Through comprehensive analysis of proteins, metabolites, and miRNAs, the team revealed that PDVs from different plant sources display significant functional variations in anti-tumor activity and tissue repair, with ginger EVLP demonstrating particularly strong anti-tumor effects. In the context of engineered preparation, Professor Li described a scalable extrusion-based method for producing PDVs, confirming their considerable potential in viral neutralization - such as against SARS-CoV-2 - and in promoting wound healing; these engineered PDVs also show low retention risk and no tumor-promoting properties. Furthermore, the study examined the functional heterogeneity of components in bacterial EVs, reinforcing the notion that the biological functions of EVs are primarily dictated by their molecular cargo, thus offering novel insights for advancing therapies in oncology and regenerative medicine.

**Professor Jiawen Wang** (Changchun University of Chinese Medicine) introduced the multiple mechanisms of action of ginseng EVLP in colitis and wound healing. Studies have shown that in experimental colitis models, ginseng EVLP alleviates macrophage oxidative stress and inflammation by activating the P62-NRF2 pathway, repairs the intestinal barrier by up-regulating tight junction proteins and promoting stem cell proliferation, and regulates the structure of the microbiota, synergistically alleviating colitis through multiple pathways. In wound healing, ginseng EVLP promotes collagen production and angiogenesis by activating the ERK pathway, accelerating the repair of common wounds. In diabetic wound models, it effectively promotes angiogenesis and the healing process by improving endothelial cell oxidative stress and activating the PI3K-AKT pathway. Its ability to regulate macrophage polarization further accelerates the repair process. Future research will focus on the identification of key active substances in ginseng EVLP and efforts to increase its yield and stability.

## PART 2 BASIC RESEARCH ON CHM-EVLPS

**Professor Ling Li** (Hunan University of Chinese Medicine) adopted a multi-omics strategy to systematically analyze the superiority of *Platycodon grandiflorum EVLP* and its regulatory role in the respiratory-gastrointestinal axis. The research found that *Platycodon grandiflorum* EVLP is rich in nucleic acids and proteins, with components closer to fresh products and a higher content of flavonoids. After oral administration, it shows good biocompatibility and digestive stability, and is specifically enriched in the intestine and lungs. It can alleviate lung inflammation by regulating the intestinal flora and improving barrier function. After intravenous injection, *Platycodon grandiflorum* EVLP can significantly alleviate acute lung injury and correct the imbalance of macrophage polarization. The mechanism is closely related to the up-regulation of key protein BCP and the promotion of M1 to M2 type macrophage transformation, thereby exerting anti-inflammatory and tissue repair effects. Future research will further focus on the metabolic pathways and action targets of *Platycodon grandiflorum* EVLP in lung-gut comorbidities.

**Professor Chaoxiang Chen (Jimei University)** systematically elucidated the mechanism of action of curcumin-based EVLP in combating obesity and aging. The study employed a strategy combining polyethylene glycol (PEG) precipitation, solvent extraction, and self-assembly, which significantly enhanced the drug loading capacity of curcumin and effectively addressed its limitations in poor water solubility and low bioavailability. In anti-obesity applications, curcumin EVLP activated the transient receptor potential vanilloid 1 (TRPV1) channel, promoted adipocyte browning and apoptosis, reduced lipid droplet accumulation, and restored mitochondrial morphology. Animal experiments further demonstrated that curcumin EVLP effectively suppressed weight gain, modulated gut microbiota, and decreased lipid deposition in both liver and adipose tissues. In anti-aging research, curcumin EVLP was specifically enriched in the intestines and brains - particularly in microglia - of mice with galactose-induced aging models. It delayed aging by regulating microglial lipid metabolism, reducing intracellular lipid droplet accumulation, and improving mitochondrial function. These findings from Professor Chen’s team provide critical experimental evidence supporting the development and therapeutic potential of curcumin EVLP for metabolic and age-related diseases.

**Professor Huantian Zhang** (The First Affiliated Hospital of Jinan University) focused on the common mechanisms of joint barrier imbalance in osteoarthritis (OA) and its therapeutic strategies. Professor Zhang pointed out that CX3CR1⁺ resident macrophages in the synovial lining layer of the joint are key to maintaining joint barrier function, and their dysregulation can exacerbate the infiltration of inflammatory factors and synovial inflammation. In metabolic OA, the glycolysis and cell recruitment ability of macrophages are enhanced, accompanied by the down-regulation of thioredoxin-interacting protein (TXNIP) and virus inhibitory protein, endoplasmic reticulum-associated, interferon-inducible (VIPERIN/VIP1) expression; interfering with TXNIP can further promote glycolysis and worsen inflammation. Mechanistically, the epigenetic regulator bromodomain-containing protein 4 (BRD4) affects the glycolytic reprogramming and polarization state of macrophages by regulating the expression of TXNIP and glucose transporter 1 (GLUT1), thereby driving the inflammatory process. Based on this, the team developed nanoparticle and CHM-EVLPs biomimetic delivery systems that can target BRD4 inhibitors to the synovial tissue, effectively inhibiting M1 macrophage polarization and glycolytic activity, thereby alleviating OA inflammation and cartilage damage. This study provides a new strategy for CHM-EVLPs to target glycolysis to restore joint barriers and delay OA progression.

**Professor Fengxi Yang** (Institute of Environmental Horticulture, Guangdong Academy of Agricultural Sciences) systematically introduced the medicinal functions of orchids and the application prospects of their EVLPs. She pointed out that orchids are rich in species, and species such as *Dendrobium, Ginseng*, and *Arundina graminifolia* exhibit multiple pharmacological activities such as antioxidant, anti-tumor, and antibacterial properties. Her team has established an extraction technology platform for orchid plant EVLP and achieved efficient and large-scale preparation through size-exclusion chromatography. Research has confirmed that orchid plant EVLP contains active components such as polysaccharides, alkaloids, and flavonoids, among which *Arundina graminifolia* EVLP shows outstanding efficacy in anti-tumor, whitening, and antioxidant aspects. In the future, Professor Yang hopes to further expand the application value of orchid plants in the fields of medicine and cosmetics by strengthening their genetic breeding and industrial development.

**Professor Lifu Wang** (Guangzhou Medical University) expounded on the mechanism of action of *Coptis chinensis EVLP* in the treatment of inflammatory bowel disease (IBD). In response to the current clinical predicament of significant drug side effects and high postoperative recurrence rates in IBD treatment, his team discovered that *Coptis chinensis* EVLP can effectively alleviate the symptoms of colitis in mice and maintain the integrity of the intestinal barrier by promoting the proliferation and repair of intestinal epithelial cells and inhibiting cell apoptosis. Mechanistic studies have shown that *Coptis chinensis* EVLP delivers miRNA-5106 to target and regulate the expression of solute carrier family 39 (SLC39), thereby inhibiting the formation of neutrophil extracellular traps (NETs) and ultimately reducing intestinal inflammatory responses, without observing any obvious toxic side effects. This research provides new potential strategies and theoretical basis for the clinical treatment of IBD.

**Professor Han Liu** (Shanghai University Institute of Translational Medicine) reported on the research progress of PDVs in the field of translational medicine for bone aging. He pointed out that EVs, as a new type of nanocarrier for drugs, are expected to break through the current research bottlenecks of bone aging-related diseases. In response to the problems of limited production and single function of traditional EVs, the team developed new high-yield and multi-functional EV materials derived from plants and organoids. Based on synthetic biology strategies, the team successfully constructed engineered EVs from probiotics, which can precisely regulate the activity of osteoblasts and osteoclasts, significantly improving the treatment effects of osteoporosis and OA. In addition, the team developed a probiotic EV composite hydrogel system, which can effectively promote bone tissue repair by delivering anti-inflammatory and osteogenic active factors. Professor Liu also proposed the new concept of “Organoid Tissue Extracellular Vesicle” (OTEV), providing a new research tool for simulating the real microenvironment *in vivo*. This series of interdisciplinary research provides efficient and safe new strategies for regenerative medicine in the precise intervention of bone aging, and strongly promotes its clinical application.

## PART 3 RESEARCH AND APPLICATION OF EVLPS DERIVED FROM FRUITS

**Professor Junbing Fan** (School of Basic Medical Sciences, Southern Medical University) reported that his team has developed an innovative tumor delivery system based on fruit EVLPs. Professor Fan highlighted that drug delivery in solid tumor therapy faces significant challenges, including low delivery efficiency, limited deep tumor penetration, and the development of drug resistance. The study leveraged natural bioactive components - such as vitamin C and nobiletin - abundant in fruit EVLP, and integrated them with biomimetic delivery technology to enhance drug delivery efficiency through molecular recognition and morphological adaptability. Experimental results demonstrated that lemon EVLP loaded with a low dose of doxorubicin not only induced tumor cell apoptosis and potentiated immunotherapy efficacy but also reversed multidrug resistance and significantly prolonged animal survival. Furthermore, the team developed microdroplets and macroscopic embolic agents tailored for interventional liver cancer therapy, enabling precise embolization of tumor vasculature. This work presents a novel strategy for the application of fruit EVLP in oncology.

**Professor Suhua Qi** (Xuzhou Medical University) presented on the bitter gourd EVLP and its role in the treatment of diabetes. Her team’s research found that bitter gourd can regulate key metabolic enzymes such as glycogen synthase and phosphorylase, and activate the PI3K-AKT pathway to maintain blood glucose homeostasis. In two types of diabetic mouse models, its hypoglycemic effect was superior to that of metformin, and there was no significant rebound in blood glucose after drug withdrawal. The team further identified the bitter gourd polypeptide as the key active component, which can effectively promote insulin secretion and participate in tissue repair. The related achievements have been patented, and plans are in place to promote clinical transformation, providing a new natural drug strategy for the treatment of diabetes.

**Professor Wei Yang** (Tongji Medical College, Huazhong University of Science and Technology) developed a composite system based on wolfberry EVLP and fibrin gel and systematically explained its mechanism of action in myocardial infarction repair. The study selected fibrin gel with good biocompatibility as a carrier, which not only enhanced the adhesion and retention time of EVLP in myocardial tissue but also provided three-dimensional structural support, thereby prolonging the biological effect. Under hypoxic conditions, this composite system significantly increased the survival of myocardial cells and inhibited apoptosis. Animal experiments further demonstrated that wolfberry EVLP-gel could significantly improve the cardiac function of mice with myocardial infarction, delay the death process, and the repair effect could last up to 28 days after intervention. Mechanistically, wolfberry EVLP inhibited the activation of the P38 and nuclear factor kappa-B (NF-κB) pathways, reducing myocardial fibrosis and cell apoptosis; lactose and arabinose contained therein were identified as key active components, which synergistically promoted myocardial regeneration and angiogenesis. This study not only confirmed the application potential of wolfberry EVLP in myocardial infarction repair but also provided experimental evidence for the transformation of food and medicine homologous molecules in regenerative medicine.

**Professor Xiaozhou Mu** (Clinical Medical Research Institute, Zhejiang Provincial People’s Hospital) introduced a new oral anti-inflammatory strategy using honeysuckle EVLP. His team extracted EVs using phase separation flow filtration technology and found that they have a spherical structure, high stability, and rich bioactive components, making them suitable for oral administration. Experiments showed that honeysuckle EVLP exhibited significant anti-inflammatory effects in various inflammatory models such as IBD, diabetic foot, and acute lung injury, reducing tissue damage, regulating immune function, promoting the recovery of intestinal barrier function, and further inhibiting inflammation by regulating the intestinal microbiota and metabolite levels. Professor Mu also demonstrated the application potential of honeysuckle EVLP in multiple fields including non-alcoholic fatty liver disease, lung injury, cardiac toxicity relief, and antiviral infection. His team plans to establish a PDVs database and combine it with intestinal microbiota research to explore its wide application in disease treatment.

**Professor Xiaohong Yang** (Chongqing Institute of Green and Intelligent Technology, Chinese Academy of Sciences) developed a hybrid-mode microsphere purification material in response to the clinical predicament where drug-resistant bacteria such as Methicillin-Resistant *Staphylococcus aureus* (MRSA) survive within host cells and are difficult to be effectively eliminated by traditional antibiotics. This material achieves rapid and efficient purification of PDVs through the synergistic effect of a hydrophilic shell and a hydrophobic core. The team successfully extracted high-purity and low-cost EVLP from *Toona sinensis*, which is rich in antibacterial active components and can rapidly disrupt the integrity of MRSA cell membranes, and exhibits a synergistic antibacterial effect with vancomycin. Further, through the design of a mannose-modified iron carrier, the system achieved dual-targeted delivery to macrophages and pathogenic bacteria, significantly enhancing the antibacterial efficiency. The *Toona sinensis* EVLP has good biological safety, with no obvious toxicity or hemolytic reaction. It has now been scaled up for automated production and shows clear clinical translation prospects in addressing antibiotic resistance.

**Professor Xiangsheng Cai** (Guangzhou Eleventh People’s Hospital) explored the application of corn silk EVLP in hyperuricemia. He pointed out that traditional uric acid-lowering drugs have liver and kidney toxicity and gastrointestinal side effects, while corn silk EVLP, which is of the same origin as both medicine and food, is an ideal alternative due to its natural source and multiple activities. The team successfully extracted corn silk EVLP through wall-breaking technology and confirmed its significant uric acid-lowering effect in a mouse model of hyperuricemia. Mechanism studies indicated that corn silk EVLP may exert therapeutic effects through multiple pathways by regulating the expression of uric acid metabolism-related genes such as ATP-binding cassette subfamily G member 2 (ABCG2), urate transporter 1(URAT1), and GLUT, and by coordinating the regulation of intestinal flora and metabolites. This research provides a safe and low-cost new intervention strategy for hyperuricemia and also expands a new direction for the modern application of “food and medicine of the same origin” materials.

## PART 4 CHM-EVLPS AND MEDICAL LABORATORY TESTING

**Professor Sergio Bernardigi** (University of Rome Tor Vergata) systematically reviewed the significant evolution of laboratory medicine from traditional manual operations to automation and then to digitalization, emphasizing its core position in the modern medical system. He pointed out that laboratory medicine is undergoing a fundamental transformation from manual operation to automation and digitalization, and its role is shifting from passive support to active health management. Although AI is still rarely applied in clinical laboratories, it has great potential in data processing and predictive analysis; point-of-care testing, despite facing quality control challenges, is indispensable in emergency and chronic disease management. He stressed that laboratory medicine needs to collaborate deeply with data science and the industry to develop clinically applicable AI tools and testing equipment. In the future, large-scale platforms integrating biobanks and AI diagnostic systems will become a key force in promoting precision medicine and health management.

**Professor Qi Li** (Xiyuan Hospital, China Academy of Chinese Medical Sciences) systematically expounded on the key role and development challenges of AI in the integration of TCM and laboratory medicine. He pointed out that digitalization is the foundation for the application of AI. Only by converting real-world information into structured data can algorithms effectively my patterns. In medical practice, AI significantly enhances the efficiency of laboratory tests through technologies such as image recognition and natural language processing, enabling automatic result review and disease model construction. In the field of scientific research, AI tools can accelerate literature screening, data processing, and experimental simulation. In the study of TCM, AI focuses on promoting the standardization of syndromes and the construction of an efficacy evaluation system. Through big data analysis, it establishes correlation models between syndromes and laboratory indicators, optimizing the evaluation of TCM efficacy. Professor Li also warned of the potential risks brought by AI, including damage to academic originality, the degradation of thinking ability, and ethical issues, emphasizing the need to pay attention to its negative impacts while promoting technological application.

**Professor Canyun Xu** (Ji’an Central People’s Hospital) expounded on the multiple mechanisms of action and application prospects of cordycepin in neurological diseases. Research shows that cordycepin can enhance synaptic plasticity and promote neuronal communication, thereby improving learning and memory abilities; in Parkinson’s disease models, it can regulate the activity and metabolic products of dopamine neurons, improving behavioral functions; in Alzheimer’s disease, it exerts neuroprotective and cognitive improvement effects by regulating microglial polarization, increasing neurotrophic factors, and inhibiting neuroinflammation. Additionally, it can rapidly alleviate symptoms of depression and anxiety through regulating neurotransmitters, improving neural plasticity, and modulating the gut microbiota. Professor Xu pointed out that although cordycepin has broad neuroprotective potential, its clinical application is still limited by low bioavailability, rapid metabolism, and short half-life. Optimization through drug delivery systems and structural modifications is needed, along with more high-quality clinical trials to verify its efficacy.

**Professor Zhongfang Wang** (Guangzhou National Laboratory) presented the research progress on *in vivo* construction of Chimeric Antigen Receptor T cell (CAR-T cell) therapy based on targeted mRNA/LNP delivery technology. This technology overcomes the limitations of traditional CAR-T therapy, such as complex *in vitro* preparation, high cost, and risks associated with viral vectors, by enabling the direct generation of functional CAR-T cells *in vivo*. In the study, the team constructed a CAR/interleukin-5 (IL-5) mRNA system targeting eosinophils and a CD19 CAR mRNA system targeting B cells. Both systems achieved highly efficient and specific treatment in asthma and systemic lupus erythematosus (SLE) models: the former significantly alleviated airway inflammation, while the latter effectively cleared B cells and improved autoimmune symptoms. The *in vitro* delivery efficiency of this technology reached 50% to 80%, and 20% *in vivo*, demonstrating great potential for clinical application.

**Professor Yuanchen Ma** (Guangdong Provincial People’s Hospital) pointed out that venous thromboembolism (VTE), which includes deep vein thrombosis and pulmonary embolism, is a common and serious complication after orthopedic surgery. It is characterized by high concealment and great danger. Through typical cases, he expounded the clinical challenges of VTE and the necessity of early intervention, and systematically introduced a comprehensive prevention and control system centered on risk assessment, graded prevention, and dynamic monitoring. Among them, thrombosis markers can quickly reflect the state of coagulation and fibrinolysis, and have important value in the early identification and efficacy evaluation of VTE. Practice has shown that improving the VTE prevention and control strategy can significantly reduce the risk of its occurrence and improve the prognosis of patients. Professor Ma’s presentation provides a new perspective on the therapeutic direction of CHM-EVLPs.

**Professor Xiangdong Kang** (Shanghai University of Traditional Chinese Medicine) delved into the evolution of TCM’s theory on tumors and its integration with modern medicine. He pointed out that although TCM does not have the term “tumor”, conditions such as “zhengjia” and “yan” are highly relevant. Their pathogenesis can be summarized as deficiency of the body’s vital energy and excess of pathogenic factors, stagnation of qi and blood stasis, *etc*. The treatment focuses on strengthening the body’s vital energy and eliminating pathogenic factors. Subsequently, he used ursolic acid as an example to explain its multi-pathway anti-tumor mechanism: this component can inhibit tumor cell proliferation, promote apoptosis, inhibit invasion and metastasis in a dose-dependent manner, and down-regulate MYD88 to inhibit protective autophagy. This research provides a scientific basis for the development of anti-tumor drugs from TCM and suggests that optimizing delivery through strategies such as nanotechnology can enhance its clinical application prospects.

**Professor Honghai Hong** (Guangzhou Medical University Third Affiliated Hospital) presented the innovative application of AI technology in blood routine tests and thalassemia screening. In the field of blood routine tests, AI significantly improves the efficiency of tests by automatically identifying abnormal cell types and generating diagnostic reports, especially in the rapid marking of leukemia blast cells and immature granulocytes. In thalassemia screening, the AI model developed by the team, based on 12 parameters, achieved a sensitivity and specificity of 0.93 and 0.91, respectively, outperforming traditional methods. This model can effectively identify patients of different genders and disease states, reducing the risk of missed diagnosis and over-examination, and has been successfully applied in prenatal diagnosis and male screening. Future research will focus on achieving precise typing of α and β thalassemia and exploring the feasibility of replacing traditional electrophoresis and genetic testing.

## PART 5 CHM-EVLPS AND HIV

**Professor Tong Wang** (College of Life Science and Technology, Jinan University) conducted an in-depth investigation into the intricate interplay between EVs and HIV-associated diseases. He highlighted that HIV exploits the host’s EV machinery, leveraging its transport pathways to facilitate viral budding and transmission - particularly in T cells and macrophages. While current antiretroviral therapies effectively suppress viral replication, therapeutic strategies targeting the latent HIV reservoir remain lacking, representing a major obstacle to achieving a functional or sterilizing cure for acquired immune deficiency syndrome (AIDS). Professor Wang further identified three critical clinical challenges associated with chronic HIV infection: neurodegenerative disorders, non-AIDS-defining malignancies, and incomplete immune reconstitution. He emphasized that these conditions share a common underlying mechanism involving persistent HIV DNA transcription and chronic immune activation. Notably, in patients with incomplete immune recovery, elevated intracellular transcriptional activity continues to drive inflammation and immune dysfunction, even under sustained viral suppression. To address this challenge, Professor Wang proposed a novel therapeutic strategy utilizing CHM-EVLPs for HIV treatment. This approach holds potential for modulating macrophage-mediated inflammation and suppressing the activity of the HIV reservoir, thereby offering a promising new avenue toward more effective management of AIDS.

**Professor Xin Deng** (Guangxi University of Chinese Medicine) highlighted the distinctive role of TCM in the prevention and management of AIDS among the elderly. He noted that the incidence of AIDS in older adults in China has been steadily increasing, with sexual transmission - including same-sex transmission - now the predominant route among individuals aged 50 and above. This trend is closely associated with evolving sexual attitudes, inadequate awareness of HIV/AIDS, and the misuse of aphrodisiac drugs. Elderly individuals living with HIV often suffer from multiple chronic comorbidities and exhibit significantly compromised immune function, leading to their classification as a vulnerable population in recent clinical guidelines. Evidence indicates that TCM offers clear benefits in enhancing immune function, reducing the frequency of opportunistic infections, mitigating adverse effects of antiretroviral therapy, and improving overall quality of life in this demographic. Particularly under the guiding principle of “preventing disease before it occurs”, TCM demonstrates notable efficacy in regulating spleen and stomach functions and reinforcing spleen-kidney health. Professor Deng stressed the urgent need for more rigorous, high-quality clinical studies to generate robust evidence-based data and strengthen international academic recognition. Moving forward, further integration of TCM and Western medicine through systematic collaboration will be a key strategy in advancing precise and effective prevention and treatment approaches for elderly patients with AIDS.

**Professor Ying Liu** (AIDS Center of China Academy of Chinese Medical Sciences) introduced the research progress of TCM in the treatment of AIDS. She reviewed the history of TCM’s involvement in AIDS treatment, emphasizing its unique advantages in increasing CD4-positive T cell counts, improving clinical symptoms, reducing drug side effects, incomplete immune reconstitution, opportunistic infections, skin diseases, and mental disorders through methods such as supporting the body’s defenses and eliminating pathogenic factors, clearing heat and detoxifying. She also introduced the application and dosage form research of TCM compound prescriptions, and stressed the important role of TCM in the comprehensive prevention and treatment of AIDS. She called for the establishment of a multidisciplinary comprehensive prevention and treatment discipline and the combination of TCM with modern technology to achieve the goal of prolonging patients’ lives and improving their quality of life.

**Professor Weihua Li** (Beijing You’an Hospital, Capital Medical University) expounded on the new mechanism of TCM in treating HIV through the exosome pathway. His team discovered that active components of TCM such as astragalus polysaccharides can activate latent HIV reservoirs via exosomes, enhance the function of HIV-specific T cells, and synergistically boost the immune response. This mechanism provides precise targets for the development of TCM compound formulas and the innovation of AIDS treatment strategies, promising to break through the bottleneck of incomplete immune reconstitution and promote the formation of an “immune regulation-virus suppression” synergistic treatment model, facilitating the clinical transformation of functional cure. Professor Li also pointed out that exosome research still faces technical challenges such as separation and purification, and it is necessary to further clarify the molecular pathways by which TCM regulates exosomes, optimize dosage form design, and promote the clinical translation of exosome technology to open up new breakthrough paths for AIDS treatment.

**Professor Luping Lin** (The Eighth Affiliated Hospital of Guangzhou Medical University) discussed the clinical application and research progress of TCM characteristic therapies in the treatment of AIDS. She pointed out that TCM is not only limited to drug intervention in AIDS treatment but can also significantly improve the quality of life of patients through non-drug therapies, especially in special populations such as the elderly, drug-resistant patients, and those with neurological symptoms. Professor Lin systematically introduced the clinical application of several TCM characteristic therapies: acupuncture can effectively relieve pain, improve sleep, and regulate nerve function; acupoint application can help improve intestinal peristalsis and sleep disorders; and Chinese herbal fumigation and washing can relieve fatigue and anxiety. These therapies are all guided by TCM theory and have shown unique advantages in the management of symptoms in different systems. In addition, moxibustion has also shown potential application value in immune regulation, chronic symptom management, and viral reservoir control. Professor Lin emphasized that it is necessary to further verify the mechanisms of these therapies through modern scientific research to promote the standardization and internationalization of TCM characteristic therapies.

## PART 6 YOUTH FORUM

**Professor Zhiguo Zhang** (Institute of Basic Theory of TCM, China Academy of Chinese Medical Sciences) introduced that the *Pulsatilla chinensis* EVLP has a significant therapeutic effect on ulcerative colitis. In the mouse model induced by dextran sulfate sodium (DSS), *Pulsatilla chinensis* EVLP can effectively reduce the disease activity index, alleviate weight loss and bloody stool symptoms, and inhibit the release of inflammatory factors and promote the repair of intestinal barrier. After oral administration, *Pulsatilla chinensis* EVLP specifically accumulates in the cecum and colon regions, showing good biocompatibility and safety. Mechanistically, it improves the intestinal microenvironment by regulating the structure of the intestinal flora and reducing the abundance of pathogenic bacteria such as Bacteroides. Future research will further explore its mechanism of action to provide new strategies for the prevention and treatment of ulcerative colitis.

**Professor Yali Liu** (Nanjing University of Chinese Medicine) introduced the potential and mechanism of action of *Perilla frutescens* leaf EVLP in the treatment of psoriasis. Research shows that *Perilla frutescens* leaf EVLP can effectively inhibit the abnormal proliferation of keratinocytes and the expression of IL-17, and has anti-inflammatory and antioxidant activities. In a psoriasis mouse model, these EVs can efficiently penetrate the skin, improve inflammatory responses and barrier function, and no obvious toxicity was observed. Mechanistic studies indicate that the key active component, pab-miR396a-5p, exerts therapeutic effects by regulating the HSP90 and NF-κB signaling pathways. By combining with microneedle technology, the transdermal efficiency and the improvement of the skin thickening effect of *Perilla frutescens* leaf EVLP have been further enhanced. This study also suggests that *Perilla frutescens* leaf EVLP has application prospects in the field of cosmetics for anti-acne, anti-inflammatory, and skin repair.

**Professor Tianxin Qiu** (Third Affiliated Hospital of Guangzhou University of Chinese Medicine) presented the research results of CHM-EVLPs in alcohol detoxification and the prevention of alcoholic liver disease. Her research shows that the *Pueraria lobata* EVLP can effectively reduce blood ethanol and acetaldehyde concentrations by enhancing the activity of aldehyde dehydrogenase, significantly shorten the duration of intoxication, and promote sobriety. Moreover, this EVLP further demonstrates liver-protective functions, capable of lowering alanine transaminase (ALT) and aspartate transaminase (AST) levels, alleviating oxidative stress and mitochondrial damage, and inhibiting the NF-κB and mitogen-activated protein kinase (MAPK) pathways, thereby reducing inflammation and cell apoptosis. In a chronic alcoholic fatty liver model, *Pueraria lobata* EVLP can regulate blood lipids, improve liver-intestinal pathological conditions, and restore the balance of the microbiota. Professor Qiu pointed out that although CHM-EVLPs have significant advantages in alcohol detoxification and liver protection, they still face key challenges such as separation standardization, quality control, and industrialization.

**Professor Jiayang Gao** (The Chinese University of Hong Kong) delved deeply into the morphology and function research of PDVs. He pointed out that compared with the relatively clear classification of EVs such as exosomes, microvesicles and apoptotic bodies in mammalian cells, the research on PDVs has lagged behind due to the existence of plant cell walls; although there is controversy, more and more evidence indicates that plant cells can also secrete EVs. His research team used 3D electron tomography to identify three types of PDVs in plant root border cells: small EVs (50-200 nm, derived from the plasma membrane), medium-sized EVs (200-500 nm, containing ribosomes, possibly related to the EXPO pathway), and irregular large EVs (greater than 500 nm). The research further proposed a formation model and verified the universality of this classification in different plant tissues. Through proteomics and immunolabeling analysis, the team identified specific molecular markers such as TT8 and Penguin 1, revealing that these PDVs may participate in plant immune responses by carrying defense proteins. Professor Gao’s research provides a new perspective on understanding the morphology, origin, and function of PDVs, and reveals their potential role in cell communication and defense mechanisms.

This 2025 Global Conference on Research and Application of CHM-EVLPs has emerged as a pivotal catalyst for advancing the field, showcasing groundbreaking innovations and transformative insights that promise to reshape global healthcare. Standout breakthroughs include the development of novel extraction and purification technologies, such as enzymatic digestion, Tim-1 protein-functionalized magnetic nanoparticle separation, and phase separation flow filtration, that have overcome longstanding limitations of low yield, poor purity, and cumbersome processes, while preserving the bioactivity of CHM-EVLPs.

Engineering modifications, including calcium-dopamine functionalization, DNA aptamer targeting, and stress-induced remodeling of lipid membranes, have significantly enhanced the stability, targeting precision, and therapeutic efficacy of these vesicles, enabling their application in treating tumors, IBD, diabetes, and myocardial infarction. Notably, interdisciplinary integration has driven remarkable progress: the fusion of CHM-EVLPs with microneedle delivery systems, oral nanomedicine platforms, and AI-assisted drug design has expanded their clinical applicability, while insights into cross-kingdom communication mechanisms and the identification of key active components have laid a solid scientific foundation for their mechanisms of action.

Additionally, innovative explorations into HIV treatment, anti-aging interventions, and EVs have opened new frontiers, bridging TCM with modern biomedicine. Collectively, these cutting-edge reports not only addressed critical challenges such as standardization, large-scale production, and regulatory bottlenecks but also fostered global collaboration, propelling CHM-EVLPs toward clinical translation and industrialization, and marking a significant leap forward in realizing their potential to advance global pharmaceutical development and healthcare innovation.

## DECLARATIONS

### AI and AI-assisted tools statement

During the preparation of this manuscript, the AI tool Grammarly (version 1.2.228.1813, released 2026-01-28) was used solely to improve spelling, grammar, clarity, and readability. The tool did not influence the study design, data collection, analysis, interpretation, or the scientific content of the work. All authors take full responsibility for the accuracy, integrity, and final content of the manuscript.

### Copyright

© The Author(s) 2026.

